# Super-resolution microscopy: a brief history and new avenues

**DOI:** 10.1098/rsta.2021.0110

**Published:** 2022-04-04

**Authors:** Kirti Prakash, Benedict Diederich, Rainer Heintzmann, Lothar Schermelleh

**Affiliations:** ^1^ Integrated Pathology Unit, Centre for Molecular Pathology, The Royal Marsden Trust and Institute of Cancer Research, Sutton SM2 5NG, UK; ^2^ Leibniz Institute for Photonic Technology, Albert-Einstein-Strasse 9, 07745 Jena, Germany; ^3^ Institute of Physical Chemistry and Abbe Center of Photonics, Friedrich-Schiller-University, Helmholtzweg 4, 07743 Jena, Germany; ^4^ Department of Biochemistry, University of Oxford, Oxford OX1 3QU, UK

**Keywords:** super-resolution microscopy, structured illumination microscopy, computational imaging, frugal microscopy, spatial resolution, image processing

## Abstract

Super-resolution microscopy (SRM) is a fast-developing field that encompasses fluorescence imaging techniques with the capability to resolve objects below the classical diffraction limit of optical resolution. Acknowledged with the Nobel prize in 2014, numerous SRM methods have meanwhile evolved and are being widely applied in biomedical research, all with specific strengths and shortcomings. While some techniques are capable of nanometre-scale molecular resolution, others are geared towards volumetric three-dimensional multi-colour or fast live-cell imaging. In this editorial review, we pick on the latest trends in the field. We start with a brief historical overview of both conceptual and commercial developments. Next, we highlight important parameters for imaging successfully with a particular super-resolution modality. Finally, we discuss the importance of reproducibility and quality control and the significance of open-source tools in microscopy.

This article is part of the Theo Murphy meeting issue 'Super-resolution structured illumination microscopy (part 2)'.

## 1. A brief history of super-resolution microscopy

**Figure 1.**
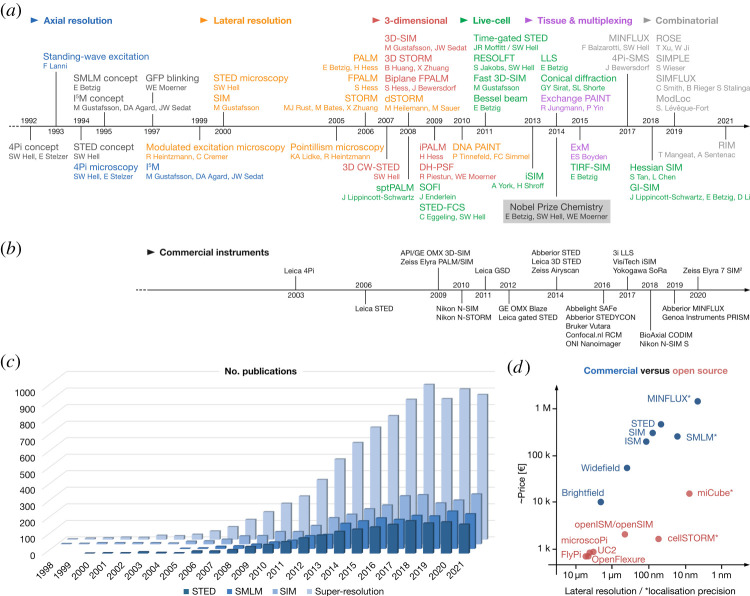
A brief history of far-field SRM. (*a*) Timelines highlighting crucial milestones in the development of SRM techniques (top) and the corresponding introduction of commercial turn-key super-resolution systems (bottom). For each SRM technique, the corresponding authors of the subsequent publication have been listed. (*c*) The number of publications extracted from the Web of Science database. (*d*) Correlation between the approximate purchase costs and the nominal lateral (*xy*) resolution or localization precision (SMLM-based systems) achievable under ideal conditions, for commercial and open-source microscopy systems, respectively. (Online version in colour.)

Optical fluorescence microscopy is a key method in modern biological and biomedical research. However, it has for the longest time suffered from the fundamental limitation of optical resolution imposed by the numerical aperture (NA) of the objective and the wavelength of light following fundamental laws of diffraction as described by Ernst Abbe [1]. Electron microscopy on the other hand, despite its ability to achieve orders of magnitude better resolution, has traditionally been hampered by the challenges in sample preparation and the difficulty in labeling and identifying specific molecules and structures, as well as a very low throughput. Hence physicists sought for ways to overcome the resolution barrier and bridge the gap between light and electron microscopy. Early works were confined to near-field scanning methods which had only very specialized and limited applicability in biology [2]. The first basic concepts to surpass the optical diffraction limit in far-field fluorescence microscopy were conceived in the early 1990s (see timeline in figure 1*a*). Along with optical sectioning through out-of-focus light rejection, slight improvements in the lateral resolution were already achieved by confocal microscopy [3]. Early super-resolution microscopy (SRM) developments initially addressed the inferior axial (*z*) resolution in far-field epifluorescence microscopy to achieve close to isotropic three-dimensional resolution by standing wave illumination [4] using opposing objectives in confocal (4Pi) [5,6] or widefield (I5 M) [7] microscopy. In the lateral direction, the theoretical foundations of stimulated emission depletion (STED) [8–10], structured illumination microscopy (SIM) [11–14] and single-molecule localization microscopy (SMLM) [15,16]. Yet it required the maturation of underlying laser, photo-detector and computer technology to trigger the ‘Cambrian explosion’ of practical implementations of SR techniques seen a few years later in the 2000s [17–23]. For SMLM, the *d*STORM variant, which works with conventional fluorophores, became most popular [24,25]. At this point, three major SRM techniques were established, i.e. STED, SIM and SMLM (now the widely accepted umbrella term for a raft of techniques that essentially follow the same principle and only distinguished themselves by the mechanism of stochastic switching of dye excitation states; see also table 1).

**Table 1.** Overview of major SRM techniques and their characteristics.

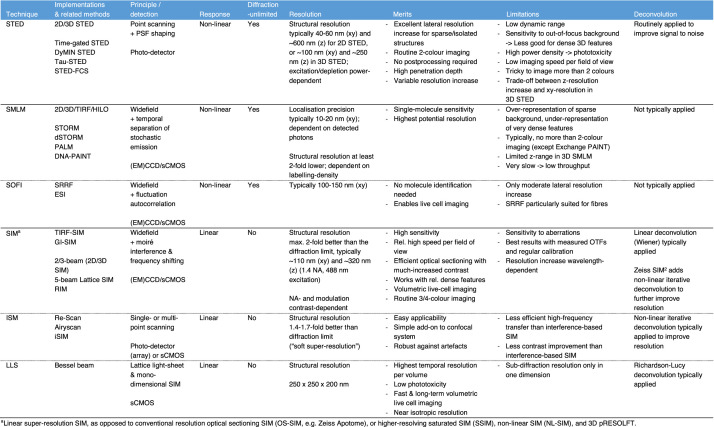

After ‘overcoming’ the fundamental limits of optical resolution, subsequent developments were mainly driven by the demands of biologists, that is to (i) enhance resolution in all spatial dimensions and/or enable volumetric imaging [26–35], (ii) improve temporal resolution and reduce photodamage at the expense of spatial resolution to enable live-cell SR imaging [36–51], and (iii) increase imaging depth and penetration for three-dimensional tissue imaging [52–57]. As a logical consequence, commercial implementation into turn-key instruments followed with a few years' delay (figure 1*b*), as did the widespread application as seen by the development of the number of publications (figure 1*c*).

The latest development push has come from combining SMLM with illumination principles of other SRM techniques, like 4Pi single-molecule switching (4Pi-SMS) [58], MINFLUX [59] and SIMFLUX type methods [60–63] to further increase either lateral or axial localization precision (figure 1*a*). Most recently, in random illumination microscopy (RIM), principles of SIM and SOFI are combined to enable 3D SRM in a more robust and user-friendly way that is less affected by aberrations [64]. In table 1, we provide an overview of major SRM techniques and their characteristics. For a more in-depth explanation of their functioning, we would like to refer to recent reviews [65–67]. Readers are also pointed to the following for further reference on this topic [68–72].

## 2. New avenues for super-resolution microscopy

No matter what leads to the development of a particular microscopy technique, whether it is the curiosity to build something new, to achieve the highest possible resolution, to gain new insights in natural, material or other sciences, to prove new physical phenomena or simply the joy of the technical interaction between man and machine—microscopy is almost always the result of many different disciplines working together. Nobel Prize-winning methods such as Cryo-EM, PALM or STED show that with the combination of new technology, the clever use of photophysical dye properties or computational tools, the optical resolution limit once described by Ernst Abbe can be circumvented.

Nonetheless, there is still plenty of room for ongoing and future improvements. Importantly, current progress is mainly geared towards promoting the reliability and applicability of these advanced techniques, rather than to further increasing resolution. On the instrument side, the wider-spread implementation of adaptive optics (AO) to correct (sample-induced) aberrations will benefit all the above-mentioned techniques to achieve their theoretical resolution in less ideal optical conditions, e.g. in thick cells and tissues [73–78].

Major advances are expected from the development of correlative microscopy approaches that seek to combine the strengths of different complementary imaging techniques [79–81]. For instance, recent advances in super-resolution cell imaging under cryo-conditions [82–84] and three-dimensional electron microscopy using advanced FIB-SEM [85] open up a potential pathway for developing new powerful three-dimensional CLEM workflows. Also, the consecutive application of SIM and single-molecule imaging techniques (SMLM, SPT) on the same widefield imaging platform holds the promise of getting the best of two worlds: single-molecule localization and contextual and structural three-dimensional information from SIM.

Major progress has been and continues to be provided by improvement in fluorescent dyes, probe design and labelling tools, such as in DNA-PAINT [86], Halo-tag JF dyes [87,88] and nanobody reagents [89]. These have been specifically designed with SRM in mind, to improve specificity and photon yield and live-cell imaging or, in combination with microfluidics or waveguide-based SRM imaging, enable multiplexing for future development [90–93].

Any progress on the instrument and labelling side will be accompanied by the massive utilization of bespoke Artificial Intelligence (AI) enhanced software solutions, implementing machine learning or neural networks to simplify and improve data post-processing/analysis [94,95] and bridging the gap to electron microscopy by correlative (cryo) super-resolution CLEM [96]. Accelerating data acquisition speed for fast, long-term imaging will be vital as the field moves towards live imaging [57,97–99]. A last important aspect is to continue to better understand the artefacts and limitations of individual SR approaches [100–102] and educate current and future users of SRM. A broader discussion of frequently asked questions in SRM can be found here [103–105].

## 3. Biological application of SRM - what have we learned?

Still, to this date, many SRM publications are generated in specialist physics/optics labs. These often display images of previously well-characterized macromolecules or biological structures, such as microtubules, nuclear pores, or actin filaments, as examples for biological applications. This led to the widespread impression that the applicability of SRM is somewhat limited, provoking the question ‘What have we learned from SRM?’ On closer examination, this is a misperception and SRM has become a genuine tool for discovery. However, what has also become clear over the past years is that, despite the promises of microscopy companies, most SRM is still not yet ‘turn-key’ in the same way conventional widefield and confocal microscopy is today. Particular expertise is required, not necessarily in how to ‘press the right buttons’ on any given commercial system, but for the typically more complex experimental design, the higher demands on the quality of sample preparations, the more delicate system calibration, and the complexity in data postprocessing and quantitative analyses. Therefore, the amount of time and commitment required to do SRM meticulously has been (and still is) a barrier for many biological and biomedical labs to move into this field. Recognizing this fact, the establishment of centralized core facilities has become a popular path to not only make advanced imaging systems available to a wider number of research labs but also to provide the expertise to run those systems effectively. Secondly, microscopy companies and developers are meanwhile turning their efforts into making SRM more accessible and reliable, which explains the success of ‘soft super-resolution’ methods, like rescan confocal, photon reassignment, Airy scan or iSIM [36,106,107], that come as easy-to-apply add-on features to standard confocal systems.

It is also important to realize that a biological discovery does not necessarily mean revealing a new structure. Rather than creating ‘nice-looking’ pictures (although that might be a pleasant side effect), the aim of an imaging experiment is rather to generate meaningful and reproducible quantitative data that helps to explain a biological phenomenon. Here, the ability to resolve events in time is as important as distinguishing objects or (macro)molecules in space as is their relationship to other molecules and structural features. High(er) throughput/content, along with elaborate data analyses, are becoming increasingly important for cutting-edge research involving SRM, and whatever the new findings, these need then be confirmed with orthogonal methods. Ideally, super-resolved images and data spark researchers to think differently about their particular biological problem and to question long-held assumptions.

With over a thousand SRM papers meanwhile published each year (figure 1*c*), it becomes increasingly difficult to pick out highlights of new discoveries, without doing injustice to many others. Good examples can be found in the field of chromatin and RNA biology, where single-cell 3D-SIM and SMLM studies lead to fundamentally new insights and models on how nucleosomes assemble higher-order structures and topological domains to define the functional modulus of genome organization [108–113], the crucial involvement of nanodomain formation in DNA repair [114], or how Xist RNA molecules spread during X-chromosome inactivation [115]. Larger macromolecular structures and enzyme complexes, such as synaptonemal complexes, centrosomes, kinetochores, DNA repair complexes, cytoskeleton, subcellular organelles, etc. lend themselves particularly well to super-resolution studies (for recent reviews see [65,116]).

After more than one decade of development, SRM has shown that different modalities have different biological application areas and specific sweet spots of individual methods are complementary to each other. However, there is still a prevalent lack of understanding of the general benefits and limitations of one method over the other, that goes beyond comparing nominal resolution numbers (see table 1). This applies e.g. to the crucial trade-offs in SRM often depicted as ‘magical tetrahedron’ of spatial resolution, temporal resolution, photodamage, and imaging depth. Beyond this, there are further less-known trade-offs. For instance, in SMLM, the ability to localize molecules with high precision does not necessarily enable the ability to visualize and resolve macromolecular structures with enough sampling density. Increasing precision can come at the cost of decreased probability to detect localizations [69]. Moreover, both SMLM and STED employ non-linear excitation/detection to become diffraction-unlimited which comes at the price of undercounting fluorescent signals/molecules in some areas of the sample while overestimating them in others. In contrast, in linear SIM relative intensities between features with fewer or higher labeling densities are retained, allowing valid intensity quantifications. Yet this advantage gets lost when non-linear iterative three-dimensional deconvolution is added on top, which renders the data useless for particular analyses as well as generating oversharpening artifacts.

Besides biological research, SRM also has the potential to be applied in other fields, such as clinical diagnostics, e.g. using SIM through the eye lens to image the human retina with increased detail [117], or in food research using AO-assisted SMLM to investigate the characteristics of oil droplets in emulsions [118].

## 4. Open technology developments for super-resolution microscopy

Historically, SRM developments have not been open-source, e.g. due to the demand for commercialization. However, more recently the philosophy of ‘opening up’ development projects to the entire research community and harnessing communal powers to accelerate progress, has gained traction. Projects like the Openflexure Microscope [119], the Fiji Image Analysis Software [120], the cellphone-based SMLM set-up ‘cellSTORM’ [121], the three-dimensionally printed modular toolbox UC2 [93,122]) demonstrate the importance of an open and active community for scientific discovery and collaboration. Users can use, modify and build on top of existing solutions [123–125] to acquire new data, analyse them and propose new theories or questions for future generations of scientists.

The sharing of data and resources has the immense advantage of enabling other researchers to reproduce the results or even recreate entire experiments. Hence, it picks up on a currently widely discussed debate: the reproduction crisis and the associated loss of society in scientific work. On top of open-source, ‘frugal science’ aims to make scientific instruments available at low to no costs. The core idea is to replace complex and usually expensive laboratory-grade devices with do-it-yourself or consumer-grade solutions. Wang *et al*. [122] succeeded in this in the manuscript ‘UCsim2: 2D Structured Illumination Microscopy using UC2’, in which the open-source three-dimensionally printed optics kit ‘UC2’ (You.See.Too.) is extended by super-resolution functionality using SIM and image scanning microscopy (ISM). The open-source documentation allows others to build a device themselves, e.g. for teaching purposes. A device that costs only 5000€ instead of one million also contributes to the fact that several experiments are carried out in many more places in the world (figure 1*d*). This allows laboratories in financially less well-equipped institutes to keep up with cutting-edge science and contribute to scientific progress. This approach also demonstrates the beauty of open-sourcing ideas.

In addition to the possibility of providing other scientists with the tools necessary for research to increase the reproducibility of scientific results, corresponding quality standards are of great importance for carrying out the experiments. Several initiatives such as the QUAREP-LiMi [126,127] recently proposed guidelines for good practice in (light) microscopic imaging and sample preparation. The standards developed together with the scientific community promise increased reproducibility across laboratories.

In this special issue on SRM, we pick up on these trends, show new advances in super-resolution imaging and also show how important it can be to focus not only on optical resolution but also on the reproducibility of scientific results, how quality standards and the creation of communities can help, and how scientific communities can be brought closer together so that tools can be developed that can be used to make breakthroughs.

## Data accessibility

This article has no additional data.
